# Expert Opinion on Laparoscopic Surgery for Colorectal Cancer Parallels Evidence from a Cumulative Meta-Analysis of Randomized Controlled Trials

**DOI:** 10.1371/journal.pone.0035292

**Published:** 2012-04-20

**Authors:** Guillaume Martel, Alyson Crawford, Jeffrey S. Barkun, Robin P. Boushey, Craig R. Ramsay, Dean A. Fergusson

**Affiliations:** 1 Department of Surgery, Department of Epidemiology & Community Medicine, and Ottawa Hospital Research Institute, The Ottawa Hospital, University of Ottawa, Ottawa, Ontario, Canada; 2 Department of Surgery & Division of Clinical Epidemiology, McGill University, Montreal, Quebec, Canada; 3 Health Services Research Unit, University of Aberdeen, Aberdeen, Foresterhill, United Kingdom; University Hospital Carl Gustav Carus, Germany

## Abstract

**Background:**

This study sought to synthesize survival outcomes from trials of laparoscopic and open colorectal cancer surgery, and to determine whether expert acceptance of this technology in the literature has parallel cumulative survival evidence.

**Study Design:**

A systematic review of randomized trials was conducted. The primary outcome was survival, and meta-analysis of time-to-event data was conducted. Expert opinion in the literature (published reviews, guidelines, and textbook chapters) on the acceptability of laparoscopic colorectal cancer was graded using a 7-point scale. Pooled survival data were correlated in time with accumulating expert opinion scores.

**Results:**

A total of 5,800 citations were screened. Of these, 39 publications pertaining to 23 individual trials were retained. As well, 414 reviews were included (28 guidelines, 30 textbook chapters, 20 systematic reviews, 336 narrative reviews). In total, 5,782 patients were randomized to laparoscopic (n = 3,031) and open (n = 2,751) colorectal surgery. Survival data were presented in 16 publications. Laparoscopic surgery was not inferior to open surgery in terms of overall survival (HR = 0.94, 95% CI 0.80, 1.09). Expert opinion in the literature pertaining to the oncologic acceptability of laparoscopic surgery for colon cancer correlated most closely with the publication of large RCTs in 2002–2004. Although increasingly accepted since 2006, laparoscopic surgery for rectal cancer remained controversial.

**Conclusions:**

Laparoscopic surgery for colon cancer is non-inferior to open surgery in terms of overall survival, and has been so since 2004. The majority expert opinion in the literature has considered these two techniques to be equivalent since 2002–2004. Laparoscopic surgery for rectal cancer has been increasingly accepted since 2006, but remains controversial. Knowledge translation efforts in this field appear to have paralleled the accumulation of clinical trial evidence.

## Introduction

Laparoscopic surgical techniques were first introduced as a treatment for colorectal pathologies in 1991 [1], [2]. Since this pioneering work, an abundance of literature has detailed the application of this technology to both benign and malignant colorectal disorders [3]. In the early days, surgeons readily embraced laparoscopic surgery for benign colorectal conditions such as diverticulitis and inflammatory bowel disease, on the basis of perceived improvements in length of stay in hospital, postoperative pain, bowel function, and return to normal activities, as well as obvious cosmetic advantages [3]–[5]. On the other hand, the adoption of laparoscopic surgery to treat colorectal cancer has lagged behind that of benign conditions, and has been highly controversial over the last twenty years.

As with any malignant disorder, novel surgical technologies used to treat patients with curable colorectal cancer must be demonstrated to achieve improved or, at a minimum, equivalent survival outcomes. While surgical innovation has often relied on a trial-and-error approach, many surgeon-scientists now argue that novel technologies must be scrutinized carefully and tested using robust research methods [6]. It is widely agreed that randomized controlled trials (RCTs) provide the highest standard of evidence in evaluating healthcare interventions. In this context, it is not surprising that several RCTs comparing laparoscopic and open surgery for colorectal cancer have been conducted over the years, many of which have now yielded long-term oncologic outcomes.

In spite of an abundance of published data, both RCTs and observational studies, it remains unclear whether surgeons have adopted this novel technology on the basis of published high-level survival evidence. In this context, the objective of this work was to synthesize survival outcomes from RCTs of laparoscopic and open colorectal cancer surgery, and to determine whether expert acceptance of this technology in the literature has parallel cumulative survival evidence from clinical trials.

## Materials and Methods

The body of literature required to answer the research question was obtained using systematic review techniques. In the first part of this work, trials were identified and survival data were meta-analyzed. In the second part, review articles were identified and used to grade expert opinions regarding the acceptability of laparoscopy in treating colorectal cancer. Finally, both parts of this work were combined, by comparing accumulating survival data and expert opinions over time. A systematic review protocol was written and followed.

### Systematic Review

RCTs and review articles were included, on the basis of pre-determined inclusion and exclusion criteria. All RCTs pertained to patients with primary carcinoma of the colon or rectum of any stage. The primary intent of the citation had to address the treatment of colorectal cancer specifically. Included patients had to undergo a segmental resection of the colon or rectum by laparoscopic or hand-assisted laparoscopic surgery. The control intervention was open surgery. To be considered for inclusion, citations had to provide data on the primary outcome of overall survival. Trials not reporting overall survival were not included in the meta-analysis, but were retrieved to allow for the complete identification of RCTs.

For review papers, inclusion criteria were purposely less strictly defined so as to capture the entire spectrum of publications pertaining to the laparoscopic treatment of colorectal cancer. Acceptable review articles were narrative reviews, systematic reviews/meta-analyses, textbook chapters, and guidelines/policy statements. Reviews were included unless they were limited to single specific outcomes other than oncologic outcomes (eg. postoperative pain). All reviews addressing the surgical care of colorectal cancer in general terms were included, as a lack of discussion of laparoscopy would indicate that the authors did not consider it relevant.

A comprehensive search strategy was designed to identify both primary literature and review citations (see Text S1). This search strategy was designed to be highly sensitive, and was modified from previously published work [7]. Six major databases were searched for relevant citations from 1991–2008 (Ovid MEDLINE, Ovid EMBASE, Cochrane Library, Science Citation Index Expanded, BIOSIS Preview, and BIREME LILACS). An additional thirteen databases were also searched for relevant citations (Database of Abstracts of Reviews of Effectiveness, Heath Technology Assessment Database, NHS Economic Evaluation Database, NIHR Health Technology Assessment Programme, Trip Database, Clinicaltrials.gov, Controlled-trials.com, National Guidelines Clearinghouse, CMA Infobase: Clinical Practice Guidelines, NICE England, SIGN Scotland, NHMRC Australia, New Zealand Guidelines Group). In addition, all editions of nine major surgical textbooks published since 1991 were included for consideration (Schwartz’s Principles of Surgery, Sabiston Textbook of Surgery, Greenfield’s Surgery, Cameron’s Current Surgical Therapy, Shackelford’s Surgery of the Alimentary Tract, Mastery of Surgery, Gordon’s Surgery of the Colon, Rectum and Anus, Corman’s Colon and Rectal Surgery, and Fazio’s Current Therapy in Colon & Rectal Surgery). The reference lists of all included citations were screened to identify missing trials and reviews. No language limitation was applied to the search strategy.

**Figure 1:**
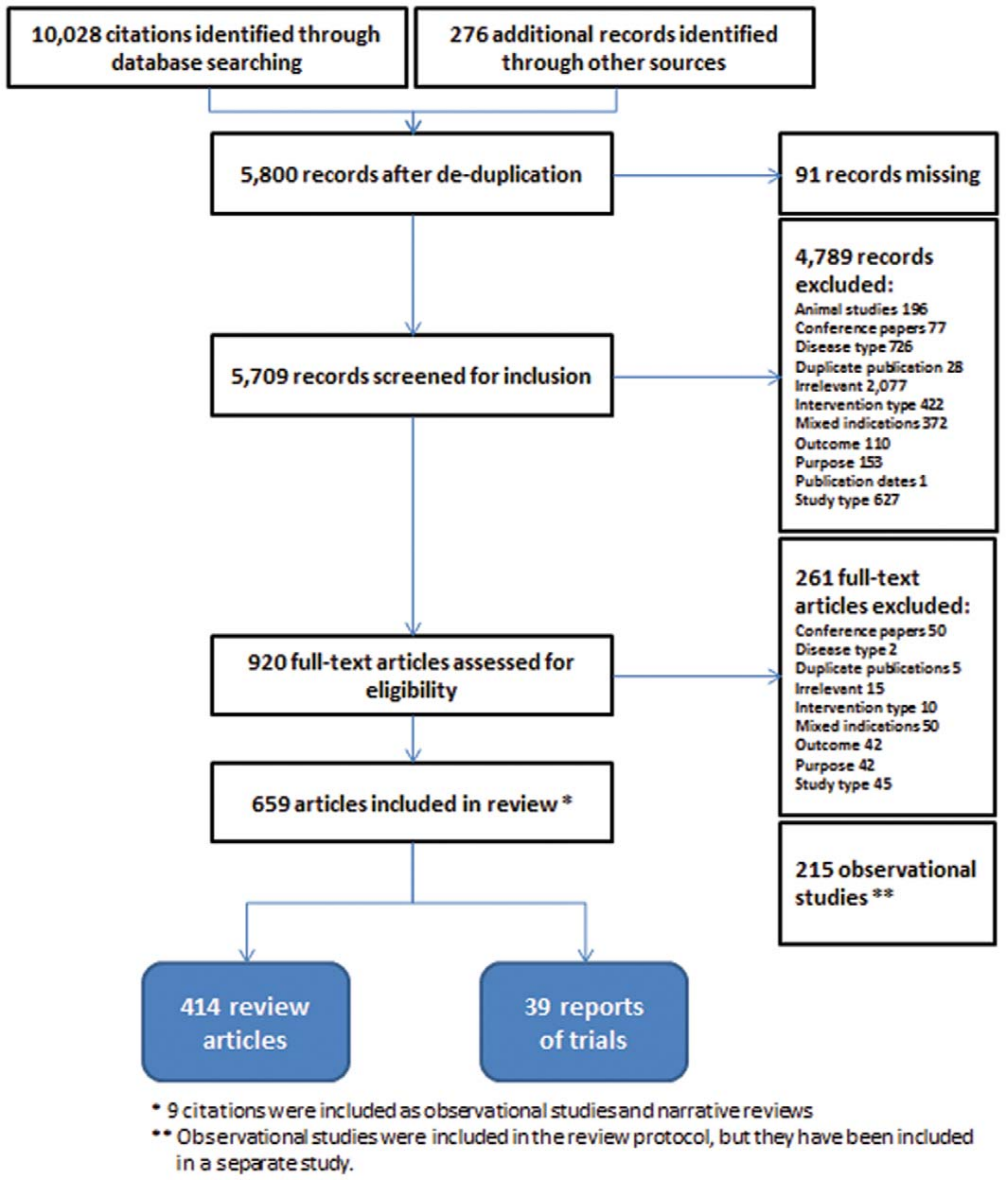
PRISMA flow diagram.

All citation records were retrieved and downloaded electronically using Reference Manager 10 (ISI ResearchSoft, Berkeley, CA), and were then de-duplicated manually. All citations were first screened for inclusion on the basis of titles and abstracts (figure 1). All retained citations were then retrieved in full text. Papers published in languages other than English, French, or Spanish were translated in full. Citations in Asian languages included after the first screen had to be excluded from further consideration due to translation resource limitations. Full-text articles were evaluated for inclusion.

Included RCTs were examined their risk of bias using the approach advocated by the Cochrane Collaboration [8]. Eight items were appraised: 1) random sequence generation, 2) allocation concealment, 3) blinding of participants and personnel, 4) blinding of outcome assessment, 5) incomplete outcome data, 6) selective reporting, 7) provider expertise bias (surgical skills acquisition and learning curves can influence outcomes in procedures-based trials [9]) and 8) other potential sources of bias (eg. randomization after carrying out a diagnostic laparoscopy).

All eight items were assessed for each trial. Where trials had more than one publication, the trial as a whole as evaluated using all included reports. As well, referenced trial protocols or partial publications not included in this review were also read in an attempt to minimize trial reporting issues. All items were graded as “low risk of bias", “high risk of bias", or “unclear risk of bias". These data were synthesized using descriptive figures. Trials with five or more items with a low risk of bias were arbitrarily defined as high-quality studies.

### Data Analysis

Data from included RCTs and review papers were extracted. Data points were checked for accuracy by a second reviewer and discrepancies were resolved by consensus. Although each citation was abstracted separately, trials with multiple publications were identified so as to avoid patient duplication. Individual trial authors were contacted selectively.

Data on overall survival were abstracted in the statistical format provided in individual reports. Relative outcomes were always recorded as a comparison of laparoscopic to open surgery. Where hazard ratios (HR) for survival and confidence intervals were not provided, available data were utilized to generate hazard ratios for each trial using published methods [10]–[12]. Briefly, this technique allows one to derive the ln(HR) and var(ln(HR)) from published statistics. Where this information is unavailable, one can utilize Kaplan-Meier curves to derive conservative estimates of HR. This method assumes that censoring is constant and non-informative across smaller time interval on the Kaplan-Meier curve (eg. 6 months). A Microsoft Excel (Microsoft Corporation, Richmond, WA) macro was utilized to facilitate all computations [12], [13].

Expert opinion in the literature was abstracted from review papers separately for colon and rectal cancers. The author’s global conclusion regarding the acceptability of laparoscopic surgery to treat colon or rectal cancer was extracted. Where multiple conclusions were provided within a single review, the expert author’s opinion regarding the primary outcome of overall survival was considered. Each review paper’s conclusion was graded on a seven-point asymmetric scale, ranging from 1, where laparoscopy was not mentioned in a review paper pertaining to colorectal cancer surgery, to 7, whereby laparoscopy was deemed the standard of care. The other options on the scale were as follows: 2) laparoscopy is inferior, 3) laparoscopy is acceptable only within clinical trials, 4) laparoscopy is equivalent with anatomic limitation, 5) laparoscopy is equivalent among experts, and 6) laparoscopy is equivalent in routine clinical practice. For data synthesis, options 1–2 were considered to indicate that laparoscopy was “inferior" to open surgery, option 3 was considered to describe clinical equipoise, while options 4–6 were deemed to indicate that laparoscopy was “equivalent" to open surgery. This scale was piloted independently by two reviewers using ten distinct review articles for ease of use and congruence among reviewers.

The primary outcome of overall survival analyzed using standard meta-analytic techniques. Derived HR were pooled using inverse variance methods and random effects models. Random effects models were preferred in this work because of the often non-standardized nature of surgery across trials, the variability in trial quality, and the added statistical conservatism provided by this approach. Where multiple publications of the same RCT were identified, only the most mature survival data were used in this meta-analysis. In contrast, a cumulative random effects meta-analysis of the primary outcome was also carried out using the earliest available survival data set for each trial, so as to assess the evolution of this outcome in time. Statistical measures of heterogeneity (Cochran’s Q and I^2^) were obtained from fixed effects models. Pre-specified sensitivity analyses were carried out based on the inclusion of colon and/or rectal cancer patients, and based on the risk of bias assigned to each trial. All analyses were carried out using Comprehensive Meta-Analysis 2.2 (Biostat, Englewood, NJ).

Grades of opinion for individual review papers were synthesized as yearly proportions, and plotted as a time series. Changing trends in expert opinions were then evaluated visually from these graphs and correlated qualitatively with overall survival data derived from RCTs, as well as compared to the temporal data obtained from the cumulative meta-analysis of overall survival.

## Results

A total of 5,800 single citations were evaluated (figure 1). After screening, a total of 38 reports pertaining to 23 individual RCTs were included [14]–[51] (see Table S1). A report of long-term data pertaining to a trial already included was also included, as it became available a few weeks after study selection [52]. In addition, 414 review citations were included, comprising 336 narrative reviews, 30 textbook chapters, 28 guidelines/position statements, and 20 systematic reviews. The full reference list is available upon request.

Among 23 individual RCTs, 5 (22%) were multicenter in design. The smallest multicenter study recruited 3 centers in Greece [41], whereas the largest multinational RCT included 48 centers across the United States and Canada [24], [26], [31], [45]. Patient recruitment varied widely, ranging from 28 patients for the smallest study from Brazil [29] to 1,082 patients for the European COLOR trial [35], [43], [52]. In total, 5,782 patients were randomized between laparoscopic (n = 3,031) and open (n = 2,751) surgery as a primary treatment for colorectal cancer. Among these patients, 58% (n = 3,336) were included as part of trials addressing only colon cancer, whereas 10% (n = 582) took part in trials limited to rectal cancer. The remainder of patients (32%, n = 1,864), were included in trials addressing colorectal cancer in general, with varying definitions as it pertains to the inclusion of rectal cancer patients.

Among the 414 review papers included in this work, 362 (87%) and 332 (80%) provided data pertaining to colon and rectal cancer, respectively. Most reviews (67%, n = 280) addressed both cancer types concurrently.

**Figure 2:**
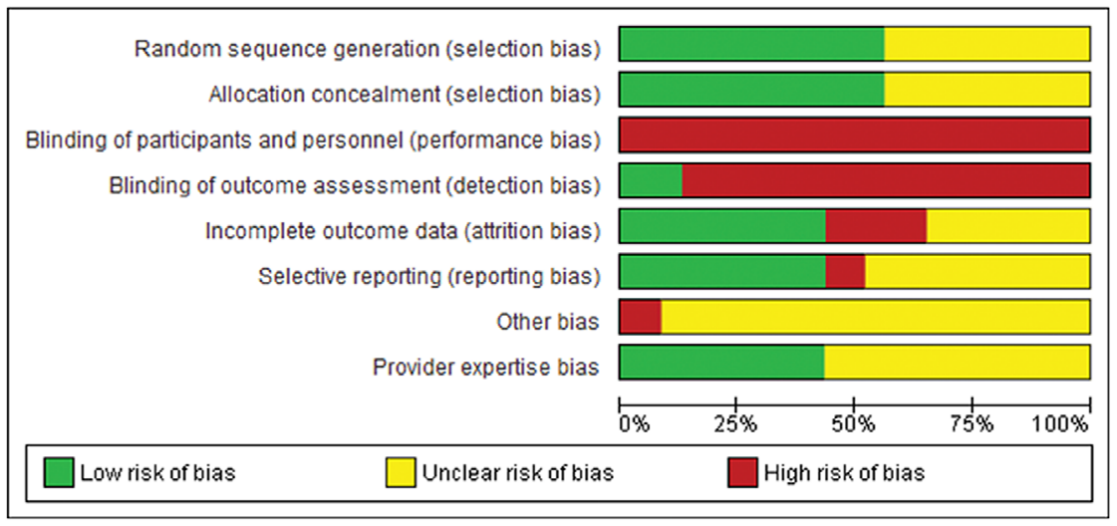
Global risk of bias assessment divided by individual items.

A total of eight risk of bias items were assessed individually (figure 2). Both items pertaining to selection bias (random sequence generation and allocation concealment), demonstrated the lowest overall risk of bias, with each item being addressed adequately by 57% of trials (n = 13), and all other trials lacking sufficient information to render judgment. Similarly, the issue of provider expertise was addressed satisfactorily by 43% of trials (n = 10), with inadequate reporting in the remainder of studies. Incomplete outcome reporting and selective reporting both also yielded a low risk of bias in 43% of trials, but these items also generated a high risk of bias in 22% and 9% of trials, respectively. In contrast, blinding – whether of patients, study personnel, or outcome assessors – was almost universally not attempted, yielding a high risk of bias in all trials except for three (13%) groups that employed blinded assessors of their primary outcomes. Two trials also demonstrated other high risks of bias, as they elected to carry out a diagnostic laparoscopy prior to randomization so as to determine whether individual patients could undergo a formal oncologic colorectal resection by laparoscopy [16]–[18], [20], [21].

Analysis of risk of bias judgments at the individual study level identified three trials that achieved “low risk of bias" marks for at least five items [24], [26], [31], [35], [43], [45], [51], [52]. These three trials were all large-scale multicenter multinational RCTs, contributing a total of 2,721 patients to this review. Seven trials obtained four “low risk of bias" marks, as certain items could not be adequately assessed from available trial reports, study protocols, or adjunct publications. Finally, 11 trials obtained a total of two or less “low risk of bias" marks, highlighting a greater overall risk of bias based on reported information. Among these, six trials obtained at least three “high risk of bias" marks [15]–[18], [20], [21], [23], [27], [28], of which five trials did not carry out intention-to-treat analyses.

**Figure 3:**
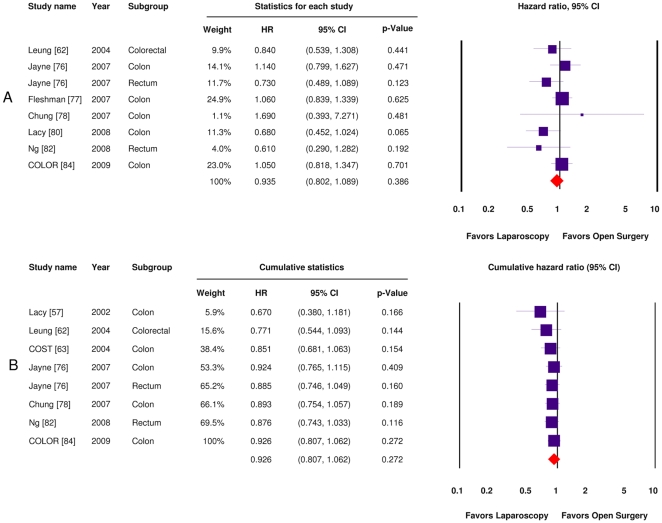
Meta-analysis of overall survival (random-effects models). **a**) Standard technique; **b**) Cumulative technique.

Data pertaining to overall survival were presented in 16 (41%) publications, originating from 13 (56%) individual trials. Median follow-up time among these trials ranged from 12–95 months. One trial did not report a median follow-up time period for either intervention group [32]. Seven trials presented sufficient data to obtain hazard ratios. Assuming a minimal clinically significant margin of 10% (HR 1.1), laparoscopic surgery for colorectal cancer was found to be non-inferior to open surgery in terms of overall survival after pooling the most mature trial data available (HR = 0.94, 95% CI 0.80, 1.09) (figure 3a). There was no evidence of statistical heterogeneity (Q = 8.996, p = 0.255; I^2^ = 22%). A cumulative meta-analysis using overall survival data as they became available in time for each trial yielded a comparable hazard ratio of 0.93 (95% CI 0.81, 1.06) (figure 3b). Stability of the pooled HR and confidence interval was achieved after publication of the COST trial in 2004 [31].

Pooling studies including only colon cancer patients (n = 5) yielded a HR of 1.01 (95% CI 0.86, 1.19), with no significant statistical heterogeneity (Q = 4.762, p = 0.313; I^2^ = 16%). The addition of a sixth trial, which included both sigmoid colon and high rectal cancers, did not alter these data significantly (not shown). Pooling trials including only rectal cancer patients (n = 2) yielded a HR of 0.70 (95% CI 0.49, 1.00), with no statistical heterogeneity (Q = 0.174, p = 0.676; I^2^ = 0%). The addition of a third trial including only rectosigmoid cancer patients to the rectal cancer group yielded a HR of 0.75 (95% CI 0.57, 0.99, I^2^ = 0%). Pooling of the two trials with the lowest risk of bias did not significantly alter the results (HR = 1.06, 95% CI 0.89, 1.25, I^2^ = 0%). Similarly, pooling all trials except one with the greatest risk of bias did not alter the outcomes, but it did increase the degree of statistical heterogeneity (HR = 0.93, 95% 0.79, 1.09, I^2^ = 28%). Pre-specified sensitivity analyses based on the risk of bias did not significantly affect the survival results or the degree of heterogeneity (data not shown).

Data on the number of lymph nodes harvested with the surgical specimen was reported by 19/23 trials (83%). Nine trials presented sufficient data to allow for meta-analysis. The range of reported lymph nodes harvested was 5.5–23.0 for laparoscopy, and 7.8–26.0 for open surgery. The pooled weighted mean difference between laparoscopic and open resection was −0.17 lymph nodes (95% CI −0.35, 0.011). This result was highly statistically heterogeneous (Q = 22.64, p = 0.004; I^2^ = 65%). A sensitivity analysis limited to colon resections yielded a similar mean difference (−0.241, 95% CI −0.632, 0.150), but with increased heterogeneity (Q = 22.44, p < 0.001; I^2^ = 82%), indicating that much of the statistical variation originates with trials addressing colon cancer rather than colorectal or rectal cancer. Analysis of rectal cancer trials (n = 2) eliminated this statistical heterogeneity (mean difference −0.129, 95% CI −0.461, 0.203, I^2^ = 0%). The addition of trials addressing colorectal cancer to those limited to rectal cancer yielded a statistically significant mean difference of −0.142 (95% CI −0.271, −0.014), with no statistical heterogeneity (Q = 0.185, p = 0.980; I^2^ = 0%). Finally, excluding high-risk of bias trials from the pooling yielded a mean difference of −0.106 (95% CI −0.211, 0.000, p = 0.05), with no evidence of statistical heterogeneity (Q = 0.974, p = 0.914, I^2^ = 0%).

**Figure 4:**
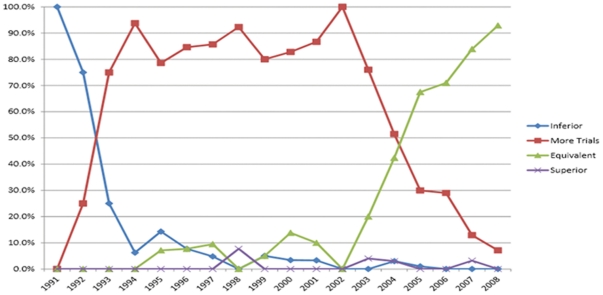
Temporal summary of expert opinion in the literature pertaining to laparoscopic surgery for colon cancer.

Distinct trends in expert opinions were identified for colon and rectal cancer. For colon cancer (figure 4), laparoscopy was initially considered inferior to open surgery, with 100% and 75% of expert opinions scoring “inferior" in 1991 and 1992, respectively. As of 1993, expert opinions curves were found to shift towards equipoise, with well over 75% of reviews opinionating that laparoscopic surgery could be considered for colon cancer in the context of prospective trials. This trend was maintained until 2003. Starting in 2003, a second major shift in opinions can be identified, as experts were found to consider laparoscopic surgery equivalent to open surgery in growing proportions. As of 2005, opinion curves were found to have crossed over, with a majority of surgeons favoring equivalency between the two technologies. After 1993, almost no surgeons considered laparoscopy inferior to laparotomy. Similarly, only four reviewers considered laparoscopy superior to open surgery at any given time point.

**Figure 5:**
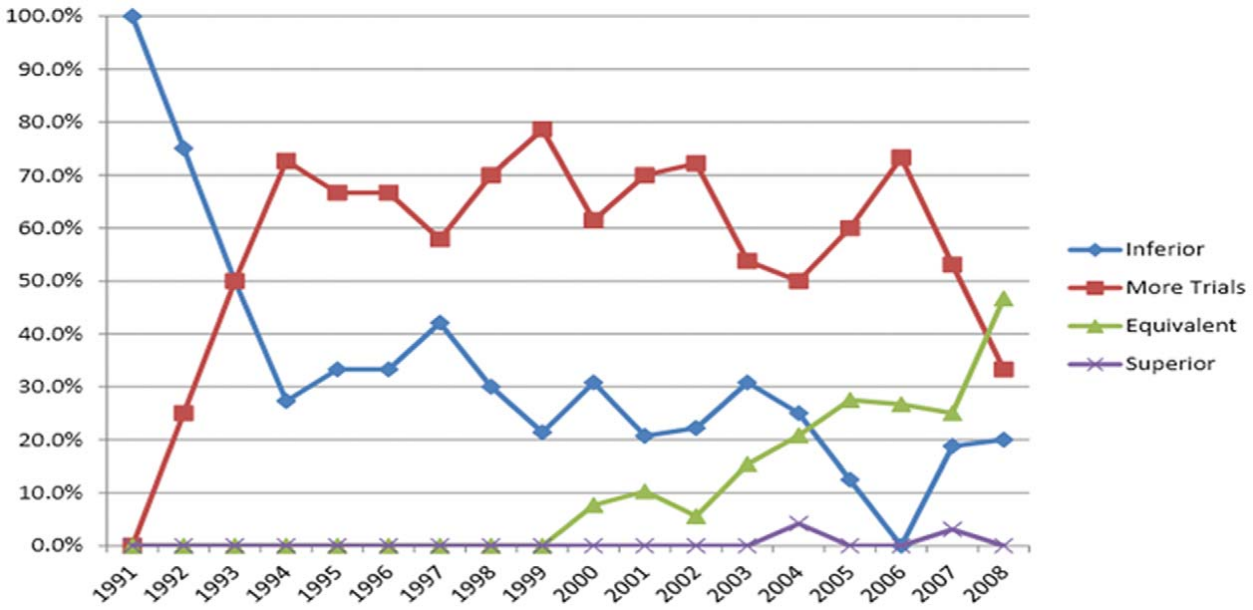
Temporal summary of expert opinion in the literature pertaining to laparoscopic surgery for rectal cancer.

For rectal cancer (figure 5), experts initially considered laparoscopy inferior to open surgery in 1991–1992. A shift towards equipoise was again noted – albeit not as strongly – from 1993–2006 with 50–78.6% of expert considering laparoscopy appropriate within clinical trials. In contrast to colon cancer, experts continued to consider laparoscopy inferior to open surgery in variable proportions (20.7–50%) from 1993–2004. A much smaller proportion of experts began to regard laparoscopy as equivalent to laparotomy for rectal cancer in 2003 onwards, although the rise was much more gradual than for colon cancer.

## Discussion

We have conducted a systematic review and meta-analysis of trials comparing laparoscopic and open surgery for colorectal cancer, addressing the primary outcome of overall survival. In addition, we have synthesized expert opinion on this topic by utilizing the entire body of relevant review literature as a surrogate for the acceptability of this technology among surgeons.

In summary, this work identified 23 RCTs presented in 39 distinct published reports comparing laparoscopic and open surgery for patients suffering from colorectal cancer. A meta-analysis of time-to-event data on overall survival demonstrated the non-inferiority of laparoscopy. Although other groups have also previously conducted meta-analyses pertaining to oncologic outcomes [53], most have opted to pool survival data using the proportion of patients alive or dead at maximal follow-up. This approach has the potential to introduce bias in a meta-analysis, as the pooling of such a dichotomous outcome involves the combination of trials at different stages of maturity and completely omits information pertaining to the timing of death following cancer surgery. In order to circumvent these issues, we have pooled hazard ratios, which are more appropriate measures of time-to-event data. Unfortunately, these data are not always reported in clinical trials. As such, we have utilized a combination of published data on hazard ratios where possible, together with best estimations of HR obtained from published survival curves. This statistical approach allowed us to generate the most comprehensive and rigorous meta-analysis of survival data pertaining to laparoscopic and open surgery for colorectal cancer available to date, as well as to confirm the non-inferiority of laparoscopy. These results compare favorably with existing meta-analyses [53], including those that employed similar statistical methods [54], [55]. The current review is also unique in its utilization of cumulative meta-analytic techniques and its comparison to expert opinion.

In addition to the above finding, we have also demonstrated that expert opinion pertaining to laparoscopic surgery for colon cancer shifted dramatically in 2003–2004. For rectal cancer, a similar – albeit weaker – trend was noted in 2006. In both instances, the majority opinion deemed that laparoscopic surgery was equivalent to open surgery after a long period of time during which this technique had reached clinical equipoise and was considered only acceptable in clinical trials. We argue that the abrupt shift in opinion in 2003 was a direct result of the publication of the first moderate-size RCT by Lacy and colleagues that presented medium-term survival data [25]. This single-center trial from Barcelona randomized 219 patients to laparoscopic and open surgery for colon cancer and argued that laparoscopy was superior to open surgery in terms of postoperative morbidity, length of hospital stay, tumor recurrence, and cancer-related survival. Overall survival was also found to be superior in an adjusted Cox model, but only trended towards significance in unadjusted data. Despite more modest long-term results [48], this paper was undoubtedly seminal in shifting expert opinion regarding laparoscopy colon cancer surgery. Finally, we also argue that this transition was further promoted by the publication in 2004 of the COST trial, for which the influence was probably greatest in North America where this trial was funded and conducted [31].

The above argument is further supported by the results of the cumulative meta-analysis for overall survival. Indeed, the accumulation of survival data presented in figure 3b demonstrates that both the magnitude and precision of the survival HR has stabilized and remained essentially unchanged since publication of the COST trial 3-year data [31]. In other words, both portions of the current study would indicate that laparoscopic surgery for colon cancer has been non-inferior to open surgery since 2004, and that the surgical literature has been supportive of this finding since 2003. Taken together, these data provide a first example of pragmatic knowledge translation in surgery based on clinical trial evidence.

Regarding rectal cancer surgery, it is not surprising that a notably weaker transition in expert opinion did not occur before 2006, as neither the Lacy [25] nor COST [31] trials included any rectal cancer patients. Although some surgeons may have extrapolated from the data on colon cancer, this is unlikely to have affected the prevailing expert opinion in the literature. In 2005, however, the CLASICC trial from the UK included 381 (48%) patients with rectal cancer [34], and it is likely that this paper had an encouraging influence on expert opinion. Although survival data from this trial were not published until 2007 [44], we argue that this publication together with mounting evidence of non-inferiority for laparoscopic colon cancer surgery began the transition for rectal cancer. That being said, since 2005, only a fraction of patients enrolled in trials of laparoscopic and open surgery have done so for rectal cancer, and it likely that many experts in the field have continued to wait for the publication of further large scale trials addressing this topic. Many surgeons argue that rectal cancer patients included in trials thus far have been carefully selected and have not been representative of challenging mid- to low-rectal lesions [50]. For this reason, it is likely that a full shift from equipoise to equivalency will await results from the ongoing COLOR II [56], ACOSOG Z6051 [57] rectal cancer trials.

There are several limitations to this study. First, our meta-analysis of survival outcomes is limited on the basis of incomplete reporting of data within primary publications. Although we have utilized statistical methods to generate estimates of hazard ratios in order to complete published data, it remains that there were relatively few trials with sufficient long-term data. A second limitation pertains to comparisons between colon and rectal cancers. Although we have pooled survival data for colorectal cancer as a whole, we have also provided sensitivity analyses to account for the important clinical differences between the two cancer types. Our analysis was limited by the relatively small number of patients with rectal cancer included in trials to date. In the absence of individual patient data, which is impractical for 23 distinct surgical trials, the methods used in the current review represent the most robust option for pooling survival data.

Limitations pertaining to our analysis of expert opinion in the literature include a certain degree of subjectivity in grading included review citations, as well as a lack of formal validation of our grading scale. We have attempted to minimize these limitations by piloting the scale prior to implementation. As well, we have constructed a scale that holds great face validity from a clinical standpoint and that reflects terminology and issues that are commonly raised in the in literature on this topic. Finally, it should be mentioned that this type of analysis and semi-quantitative correlation with our meta-analysis cannot determine with certainty the precise reason for shifts in expert opinion, as our evaluation of individual reviews did not seek to capture the reasoning behind opinions. As well, this work has sought to capture only indirect evidence of the adoption of laparoscopic surgery for colorectal cancer. Proof of adoption would indeed require data on actual uptake by surgeons rather than the utilization of a surrogate in the literature. Nevertheless, we argue that the current analysis is highly valuable as it provides a global evaluation of the state of the literature on this topic, which is presumably used by many – if not most – surgeons in evaluating novel technologies. At the very least, this analysis provides important insight into the process of knowledge translation by which a surgical technology is introduced and subsequently taken up into practice.

In conclusion, a large number of trials comparing laparoscopic and open surgery for colorectal cancer can be identified in the literature. Laparoscopic surgery for colon cancer is not inferior to open surgery in terms of overall survival, and has been so since 2004. The majority expert opinion in the literature has considered these two techniques to be equivalent, since the publication of landmark clinical trials in 2002 and 2004. Although increasingly accepted since 2006, laparoscopic surgery for rectal cancer remains controversial, likely owing to the current lack of dedicated large-scale randomized controlled trials. Knowledge translation efforts in the field of laparoscopic surgery for colorectal cancer appear to have paralleled the accumulation of clinical trial evidence.

## Supporting Information

Text S1 **Electronic search strategy.**
(DOCX)

Table S1 **Characteristics of included randomized controlled trials.**
(DOCX)
