# Supplementary material for: Expert Opinion on Laparoscopic Surgery for Colorectal Cancer Parallels Evidence from a Cumulative Meta-Analysis of Randomized Controlled Trials
Source: PLoS One. 2012 Apr 20;7(4):e35292. doi: 10.1371/journal.pone.0035292 (PMC3332109; doi:10.1371/journal.pone.0035292)
Supplement: Text S1 — Electronic search strategy. (DOCX) [file pone.0035292.s001.docx]

**SUPPORTING TEXT 1**

**Electronic Search Strategy**

***Ovid MEDLINE (1950 to July Week 4 2008)***

***Ovid MEDLINE In-Process & Other Non-Indexed Citations (4 August 2008)***

1. exp colorectal neoplasms/su

2. exp colectomy/

3. (colectom$ or hemicolect$ or colotom$).tw.

4. ((mesorect$ adj3 excision$) or (anterior adj3 resection) or abdominoperineal or proctectomy or proctocolectomy).tw.

5. or/1-4

6. exp colorectal neoplasms/

7. ((cancer or neoplas$ or adenocarcinoma$ or carcinoma$ or malignan$) adj3 (colorectal or colon$ or rectal or rectum or recto$ or sigmoid$ or instest$ or bowel)).tw.

8. or/6-7

9. adenocarcinoma/

10. carcinoma/

11. neoplasms/

12. or/9-11

13. exp colon/

14. rectum/

15. or/13-14

16. 12 and 15

17. colorectal surgery/

18. Surgical procedures,operative/

19. su.fs.

20. (surgery or surgical or surgeon$).tw.

21. resect$.tw.

22. operat$.tw.

23. or/17-22

24. (8 or 16) and 23

25. 5 or 24

26. laparoscopy/

27. Surgical procedures,minimally invasive/

28. (minimal$ adj3 (invasiv$ or access$)).tw.

29. laparoscop$.tw.

30. (key hole or keyhole).tw.

31. (hand assist$ or hand-assist$ or HALS or hand-port).tw.

32. or/26-31

33. 25 and 32

34. limit 33 to yr=1991-2008

35. animal/ not human/

36. 34 not 35

37. remove duplicates from 36

***Ovid EMBASE (1980 to 2008 Week 31)***

1. exp colon cancer/su

2. exp rectum cancer/su

3. exp colon resection/

4. exp rectum resection/

5. (colectom$ or hemicolect$ or colotom$).tw.

6. ((mesorect$ adj3 excision$) or (anterior adj3 resection) or abdominoperineal or proctectomy or proctocolectomy).tw.

7. or/1-6

8. exp colon cancer/

9. exp rectum cancer/

10. ((cancer or neoplas$ or adenocarcinoma$ or carcinoma$ or malignan$) adj3 (colorectal or colon$ or rectal or rectum or recto$ or sigmoid$ or instest$ or bowel)).tw.

11. or/8-10

12. adenocarcinoma/

13. carcinoma/

14. neoplasms/

15. or/12-14

16. exp colon/

17. exp rectum/

18. or/16-17

19. 15 and 18

20. colorectal surgery/

21. surgery/

22. su.fs.

23. (surgery or surgical or surgeon$).tw.

24. resect$.tw.

25. operat$.tw.

26. or/20-25

27. (11 or 19) and 26

28. 7 or 27

29. laparoscopy/

30. laparoscopic surgery/

31. Minimally invasive surgery/

32. (minimal$ adj3 (invasiv$ or access$)).tw.

33. laparoscop$.tw.

34. (key hole or keyhole).tw.

35. (hand assist$ or hand-assist$ or HALS or hand-port).tw.

36. or/29-35

37. 28 and 36

38. limit 37 to yr=1991-2008

39. (animal/ or nonhuman/) not human/

40. 38 not 39

41. remove duplicates from 40

***Cochrane Library (Issue 3, 2008)***

#1 MeSH descriptor Colorectal Neoplasms explode all trees with qualifier: SU

#2 MeSH descriptor Colectomy explode all trees

#3 colectom* in All Fields or hemicolect* in All Fields or colotom* in All Fields

#4 ((mesorect* NEAR/3 excision*) OR (anterior NEAR/3 resection) OR abdominoperineal OR proctectomy OR proctocolectomy) in All Fields

#5 (#1 OR #2 OR #3 OR #4)

#6 MeSH descriptor Colorectal Neoplasms explode all trees

#7 ((cancer OR neoplas* OR adenocarcinoma* OR carcinoma* OR malignan*) NEAR/3 (colorectal OR colon* OR rectal OR rectum OR recto* OR sigmoid* OR instest* OR bowel)) in All Fields

#8 (#6 OR #7)

#9 MeSH descriptor Adenocarcinoma, this term only

#10 MeSH descriptor Carcinoma, this term only

#11 MeSH descriptor Neoplasms, this term only

#12 (#9 OR #10 OR #11)

#13 MeSH descriptor Colon explode all trees

#14 MeSH descriptor Rectum, this term only

#15 (#13 OR #14)

#16 (#12 AND #15)

#17 MeSH descriptor Colorectal Surgery, this term only

#18 MeSH descriptor Surgical Procedures, Operative, this term only

#19 su.fs in All Fields

#20 (surgery OR surgical OR surgeon*) in All Fields

#21 (resect* OR operation*) in All Fields

#22 (#17 OR #18 OR #19 OR #20 OR #21)

#23 ((#8 OR #16) AND #22)

#24 (#5 OR #23)

#25 MeSH descriptor Laparoscopy, this term only

#26 MeSH descriptor Surgical Procedures, Minimally Invasive, this term only

#27 (minimal* NEAR/3 (invasiv* or access*)) in All Fields

#28 laparoscop* OR key hole OR keyhole OR hand assist* OR hand-assist* OR HALS OR hand-port in All Fields

#29 (#25 OR #26 OR #27 OR #28)

#30 (#24 AND #29)

***Science Citation Index Expanded (Web of Knowledge, 1991 to 14 August 2008)***

***BIOSIS Previews (Web of Knowledge, 1991 to 14 August 2008)***

#1 TS=(colectom* OR hemicolect* OR colotom*)

#2 TS=(mesorect* SAME excision*)

#3 TS=((colon or colorectal) SAME resect*)

#4 #1 OR #2 OR #3

#5 TS=(cancer SAME (colorectal or colon* OR rectal OR rectum OR rectosigmoid OR cecal OR caecal OR intestin* OR bowel))

#6 TS=(carcinoma SAME (colorectal OR colon* OR rectal OR rectum OR intestin* OR rectosigmoid OR cecal OR caecal OR bowel))

#7 TS=(neoplas* SAME (colorectal OR colon* OR rectal OR rectum OR intestin* OR rectosigmoid OR cecal OR caecal OR bowel))

#8 TS=(adenocarcinoma* SAME (colorectal OR colon* OR rectal OR rectum OR intestin* OR rectosigmoid OR cecal OR caecal OR bowel))

#9 TS=(malignan* SAME (colorectal OR colon* OR rectal OR rectum OR intestin* OR rectosigmoid OR cecal OR caecal OR bowel))

#10 #5 OR #6 OR #7 OR #8 OR #9

#11 TS=laparoscop*

#12 TS=(minimal* SAME (invasiv* OR access*))

#13 TS=(key hole or keyhole)

#14 TS=(hand assist* OR hand-assist* OR HALS)

#15 #11 OR #12 OR #13 OR #14

#16 (#4 OR #10) AND #15

***BIREME LILACS (16 September 2008)***

(Colorectal or colon$ or rectal or rectum or recto$ sigmoid$) and (cancer or neoplas$ or adenocarcinoma$ or carcinoma$ or malignan$) and laparoscop$

***Database of Abstracts and Reviews of Effectiveness (crd.york.ac.uk, 16 September 2008)***

Colorectal AND laparoscop*

***Health Technology Assessment Database (crd.york.ac.uk, 16 September 2008)***

Colorectal AND laparoscop*

***NHS Economic Evaluation Database (crd.york.ac.uk, 16 September 2008)***

Colorectal AND laparoscop*

***NIHR Health Technology Assessment Programme (ncchta.org, 9 September 2008)***

“Cancers” ICD Chapter Heading, by “Surgery”

***TRIP Database (tripdatabase.com, 15 September 2008)***

Colorectal cancer AND laparoscopy

***Clinical Trials (clinicaltrials.gov, 15 September 2008)***

Colorectal AND laparoscopy

***Current Controlled Trials (controlled-trials.com, 15 September 2008)***

Colorectal AND laparoscop%

***National Guideline Clearinghouse (guidline.gov, 16 September 2008)***

Gastrointestinal Neoplasms MeSH Category

***CMA Infobase (www.mdm.ca/cpgsnew/cpgs/, 16 September 2008)***

Laparoscopy OR laparoscopic

***NICE England (nice.org.uk, 16 September 2008)***

(Colorectal OR colon OR rectal OR rectum) AND Surgical procedures

***SIGN Scotland (sign.ac.uk, 16 September 2008)***

Cancer topic

***NHMRC Australia (nhmrc.gov.au, 16 September 2008)***

Health guidelines

***New Zealand Guidelines Group (www.nzgg.org.nz, 16 September 2008)***

Cancer category, surgery category

***Surgical Textbooks (all editions since 1991)***

Schwartz’s Principles of Surgery

Sabiston Textbook of Surgery

Greenfield’s Surgery: Scientific Principles and Practice

Current Surgical Therapy (Cameron)

Shackelford’s Surgery of the Alimentary Tract

Mastery of Surgery (Fischer)

Principles and Practices of Surgery of the Colon, Rectum and Anus (Gordon)

Colon and Rectal Surgery (Corman)

Current Therapy in Colon & Rectal Surgery (Fazio)
