# Supplementary material for: Expert Opinion on Laparoscopic Surgery for Colorectal Cancer Parallels Evidence from a Cumulative Meta-Analysis of Randomized Controlled Trials
Source: PLoS One. 2012 Apr 20;7(4):e35292. doi: 10.1371/journal.pone.0035292 (PMC3332109; doi:10.1371/journal.pone.0035292)
Supplement: Table S1 — Characteristics of included randomized controlled trials. (DOCX) [file pone.0035292.s002.docx]

**SUPPORTING TABLE 2**

Characteristics of included randomized controlled trials.*

| **Trial (Origin)** | **Study Years** | **Study Size**  **(Lap/Open)** | **Population** | **Providers** | **Outcomes** | **Follow-Up** |
| --- | --- | --- | --- | --- | --- | --- |
| Lacy  (Spain)  [14, 19, 22, 25, 48] | 1993-1998 | 219 (111/108)  ITT | Colon cancer, 15 cm above anal verge  Excluded: transverse colon, distant mets, organ invasion, obstruction, prior colonic surgery | Single center  Team with “wide” experience in advanced laparoscopy  Center described as “laparoscopy-devoted” | Primary: cancer-related survival  Operative  Short-term postop  Oncologic surrogates  Long-term oncologic | Median 95 months  (77-133 months) |
| Stage  (Denmark)  [15] | Before May 1996 | 34  (15/14)  Non-ITT | Colorectal cancer  Excluded: LAR, APR, signs of extensive local tumor growth | Single center  “All surgery performed by senior surgeons trained in both open and laparoscopic colorectal surgery” | Primary: not defined  Operative  Short-term postop  Oncologic surrogates | Median 14 months  (7-19 months) |
| Schwenk  (Germany)  [16, 17, 20, 21] | 1995-1996 | 60 (30/30)  ITT | Colorectal cancer (elective right hemicolectomy, sigmoid resection, AR, APR)  Excluded: rectal cancer below 12 cm from anal verge for sphincter salvage, transverse colon/flexures tumor, adjacent organ infiltration, tumor diameter >8 cm, ASA>3, flap reconstruction, BMI>32, pronounced adhesions, synchronous extracolonic tumors, uncorrectable coagulopathy, abscess or sepsis, obstruction, immunopathy, pregnancy, age<18, chronic analgesic/alcohol use | Single center  “Experienced” laparoscopic team  Same team for open and laparoscopic surgery | Primary: each reference provides different primary endpoint (pain, respiratory function, recovery of bowel function, and global quality of life), and each with sample size calculation based on different outcome but with same set of 60 patients  Operative  Short-term postop  Quality of life | Interview at 3 months |
| Milsom  (US)  [18] | 1993-1997 | 113 (59/54) Non-ITT | Colorectal cancer (right/sigmoid, upper or lower rectum), elective, curative surgery, ASA 1-3, age>18  Excluded: emergency, disseminated disease, organ invasion, tumor >8 cm, transverse/left colon tumor, middle rectum tumor, BMI>32 | Single center  Same team carried out both procedure types and postop care  Trained for several years with lap for benign disease/oncologic resections in animals and cadavers  Improved “skill and efficiency” for laparoscopy during trial period | Primary: pulmonary recovery  Operative  Short-term postop  Oncologic surrogate  Long-term oncologic | Median 20.4 months (1.5-48 months) |
| Curet  (US)  [23] | 1993-1995 | 43 (25/18)  Non-ITT | Colorectal cancer  Excluded: age<18, pregnancy, obstruction, malignant fistulization, fixation to adjacent tissue, colostomy placement/reversal alone | Single center  Attending surgeons and residents under direct supervision. All attendings had performed multiple lap-assisted colectomies for benign disease and palliation of malignancy | Primary: not specified  Operative  Short-term postop  Oncologic surrogate  Long-term oncologic | Median 4.9 years (2.5-6.3 years) |
| COST  (US)  [24, 26, 31, 45] | 1994-2001 | 872 (435/428)  Partial ITT | Colon cancer (right, left, sigmoid), age>18, able to communicate in English  Excluded: T4 or metastatic cancer, rectal or transverse colon, prohibitive adhesions, obstruction or perforation, severe medical illness, IBD, familial polyposis, pregnancy, concurrent or previous malignant tumor, ASA 4 or 5 | 48 centers in the US and Canada  Surgeons had to have performed at least 20 lap-assisted colectomies  Videotape submitted by surgeons to assess oncologic technique (mesenteric ligation, avoidance of tumor handling, identification of critical adjacent structures, thoroughness of exploration)  Ongoing random audit of tapes and assessment of bowel margins, among first 500 trial cases | Primary: tumor recurrence  Operative  Short-term postop  Oncologic surrogate  Long-term oncologic  Quality of life  Long-term other | Median 7 years (5-10 years) |
| Quah  (Singapore)  [27] | 1997-1999 | 170 (86/84)  ITT | Rectal cancer, age >18, suitable for elective surgery  Excluded: other tumor sites, contraindications to pneumoperitoneum, obstruction, malignancy in previous 5 years, synchronous multiple adenocarcinomas, pregnancy | Single center  All surgeons performing lap rectal resection were experienced laparoscopic surgeons proficient in TME | Primary: T cell count  Operative  Quality of life  Immune function (reported in separate paper, not included in this review) | Questionnaire at 1 year |
| Hasegawa  (Japan)  [28] | 1998-2000 | 59 (26/24)  Non-ITT | Colorectal cancer, preop diagnosis of T2 or T3 (N0) lesion, curative surgery  Excluded: Tis/T1 lesions (lap considered standard of care), T3 in upper or lower rectum, T3 in transverse colon | Single center  Operated on “hundreds of early colorectal cancers cases [laparoscopically] (and hence passed the learning curve)”  Started lap for Tis/T1 in 1992, when minimal risk of LN metastases. Included T2 in 1996, then T3 in 1997 | Primary: not specified  Operative  Short-term postop  Oncologic surrogate | Median 20 months (6-34 months) |
| Araujo  (Brazil)  [29] | 1997-2000 | 28 (13/15)  ITT | Rectal cancer, distal only, incomplete response to neoadjuvant therapy, staging favorable to radical resection by APR  Excluded: non specified | Single center  Some progress with the learning curve in colorectal surgery, good results with lap APR  Sufficient experience with laparoscopy that male gender, obesity, previous surgery are no longer contraindicated | Primary: not specified  Operative  Oncologic surrogate  Short-term postoperative  Long-term oncologic  Long-term other | 47.2 months |
| Leung  (Hong Kong) [30] | 1993-2002 | 403 (203/200)  ITT | Rectosigmoid cancer  Excluded: distal tumors needing anastomosis below 5 cm from dentate line, tumor >6 cm, infiltration of adjacent organs, prior abdominal operations near colorectal operation, lack of consent, obstruction, perforation | Single center  All operations done or supervised by surgeons skilled in both laparoscopic and open colorectal surgery  Most experienced with rectosigmoid tumors due to most volume | Primary: 5-year survival  Operative  Short-term postop  Oncologic surrogate  Long-term oncologic  Long-term other | Median 52.7/49.2 months (lap/open) |
| Zhou  (China)  [32] | 2001-2003 | 171 (82/89)  ITT | Rectal cancer below peritoneal reflection (1.5-8 cm from dentate line)  Excluded: other pathological types (eg. lymphoma), tumor below 1.5 cm from dentate line, emergencies, Duke D with local infiltration into other organs, no consent | Single center  Surgeons with adequate experience of open TME and laparoscopic technique    One surgeon for lap procedures (Zhou) | Primary: not specified  Operative  Short-term postoperative  Long-term oncologic  Quality of life | 1-24 months |
| Kaiser  (US)  [33] | 1995-2001 | 49 (15/20)  Non-ITT | Colon cancer (right, left, sigmoid), elective, curative, age >18, ASA 1-3, ability to participate in follow-up  Excluded: emergency, stage IV tumors, rectal or transverse colon cancer, prohibitive adhesions, ASA 4 or 5, associated GI disease (IBD, FAP), pregnancy | Single center  Surgical teams headed by 2 surgeons with “previously demonstrated extensive experience” with lap colon surgery for benign and malignant disease | Primary: cancer-specific survival  Operative  Short-term postoperative  Oncologic surrogate  Oncologic long-term | Median 35 months (3-69 months) |
| CLASICC  (UK)  [34, 36, 44] | 1996-2002 | 794 (526/268)  ITT | Colorectal cancer (right, left, sigmoid, AR, APR)  Excluded: transverse colon cancer, contraindication to pneumoperitoneum, obstruction, malignancy in past 5 years, synchronous adenocarcinoma, pregnancy, associated GI tract disease needing surgical intervention | 27 centers in the United Kingdom  32 surgeons  Both procedure types performed by same surgeon locally  Each had undertaken as least 20 laparoscopic-assisted resections | Primary: 3-year overall and disease-specific survival  Operative  Short-term postoperative  Oncologic surrogate  Long-term oncologic  Quality of life | Median 36.8/49.5 months (lap/open) |
| COLOR (Netherlands) [35, 43, 52] | 1997-2003 | 1248 (536/546)  ITT | Colon cancer, single tumor (right or sigmoid colon above peritoneal reflection), age >18  Excluded: rectal cancer, transverse colon or splenic flexure cancer, mets to liver or lung, obstruction, multiple primary colonic tumors, synchronous intraabdominal surgery, preop evidence of invasion into adjacent organs, previous ipsilateral colon surgery, previous cancer, absolute contraindication to general anesthesia or pneumoperitoneum | 29 centers in Western Europe  For lap, all surgical teams had done at least 20 lap-assisted colectomies  An unedited videotape of laparoscopic colectomy was submitted before a center participated in trial, to assess safety and thorough technique  All open cases done by team with a surgeon with credentials in colon surgery | Primary: 3-year disease-free survival  Operative  Short-term postoperative  Oncologic surrogate  Long-term oncologic  Quality of life | Median 52/55 months (lap/open) |
| Braga  (Italy)  [37, 42] | 2000-2001 ? | 391 (190/201)  ITT | Colorectal cancer, age >18, suitable for elective surgery  Excluded: cancer infiltrating adjacent organs, NYHA class >3, respiratory dysfunction (PaO2 <70), hepatic dysfunction (Child C), ongoing infection, neutrophils <2x10^9^/L | Single center  “Well-trained in both laparoscopic and open colorectal surgery” surgical team of 3 surgeons  Learning curve said to be completed before start of trial, reported in another paper (3 month trial period with lap colorectal) | Primary: postoperative complication rate  Operative  Short-term postoperative  Oncologic surrogate  Long-term oncologic  Quality of life  Long-term other | Median 54.2 months |
| King  (UK)  [38, 49] | 2002-2004 | 60 (41/19)  ITT | Colorectal cancer  Excluded: non-elective admission, preop evidence of mets, age <18, pregnancy, no consent, patient unable to have epidural anesthesia | Single center | Primary: postoperative hospital length of stay  Operative  Short-term postop  Quality of life | 12 months |
| Arteaga  (Spain)  [39] | 2003-2004 | 40 (20/20)  ITT | Rectal cancer, <15 cm from anal verge  Excluded: obstruction, perforation, preoperative diagnosis of T4 tumor, tumor >7 cm, candidate for local surgery | Single center  3 lap surgeons (2 experienced with advanced lap and open CRC surgery, and 3^rd^ one with training at centers of excellence of lap CRC surgery  All lap cases performed by 2 surgeons, team established November 2002  Lap cases performed “during the learning” curve  3 open surgeons, >5 years of experience with open rectal cancer surgery | Primary: not specified  Operative  Short-term postoperative  Oncologic surrogate | Unclear |
| Liang  (Taiwan)  [40] | 2000-2004 | 286 (135/134)  ITT | Colorectal cancer, stage 2 or 3, curative left hemicolectomy (distal transverse colon, splenic flexure, left, sigmoid colon requiring mobilizing splenic flexure), ASA 1-3, age >18  Excluded: other colorectal locations, emergency, palliation, disseminated disease or involvement of adjacent organs, mass >8 cm, BMI>40, previous major abdominal surgery | Single center  Technique for laparoscopic colon cancer surgery was well established; single surgeon  Started medial-to-lateral approach in 2000 for takedown of splenic flexure | Primary: cancer recurrence  Operative  Short-term postoperative  Oncologic surrogate  Long-term oncologic | Median 40 months (18-72 months) |
| Pechlivanides (Greece)  [41] | Unclear (before 2007) | 74 (34/39)  ITT | Low rectal cancer (distal 12 cm from anal verge)  Excluded: tumors extending to the pelvic walls or organs | 3 centers in Greece  All procedures performed or supervised by most experienced surgeon | Primary: lymph nodes harvest  Oncologic surrogate | Immediate postoperative stay |
| Chung **  (Hong Kong)  [46] | 2001-2006 | 86 (41/40)  ITT | Colon cancer (right), age >18  Excluded: no consent, unfit for surgery, emergency, obstruction, peritonitis, abscess, metastatic disease on preop work-up, synchronous tumor/polyp, tumor >6.5 cm | Single center  Two surgeons involved in all cases | Primary: postoperative pain  Operative  Short-term postoperative  Oncologic surrogate  Long-term oncolgic | Median 30/28 months (lap/open) (2-60 months) |
| Ramacciato (Italy)  [47] | 2001-2005 | 66 (33/33)  ITT | Colon cancer (right)  Excluded: obstruction, perforation, prior major abdominal surgery, clinical contraindication to surgery, infiltration of adjacent organ, more than one hepatic met >1 cm | Single center | Primary: not specified  Operative  Short-term postoperative  Oncologic surrogate | Median 6.4 months (2-60 months) |
| Ng  (Hong Kong)  [50] | 1994-2005 | 99 (51/48)  ITT | Low rectal cancer (within 5 cm from anal verge)  Excluded: tumor >6 cm, tumor infiltrating adjacent organs, recurrent disease, no consent, obstruction, perforation | Single center  Performed or supervised by surgeons experienced in both laparoscopic and open colorectal surgery | Primary: analgesic requirement and postoperative recovery  Operative  Short-term postoperative  Oncologic surrogate  Long-term oncologic | Median 87.2/90.1 months (lap/open) (22.8-150 months) |
| Hewett (Australia)  [51] | 1998-2005 | 601 (294/298)  ITT | Colon cancer (right, left, sigmoid), age >18  Excluded: advanced local disease >8 cm, mets, rectal or transverse colon cancer, emergency, BMI >35, ASA 4 or 5, associated GI disease requiring extensive operative evaluation or intervention, pregnancy, other cancer in past 5 years | 31 centers in Australia and New Zealand  “Stringent credentialing criteria”, as with COST trial  Audited videotape of lap colorectal procedure and 20 operative reports describing oncologically appropriate laparoscopic procedures  Participating surgeons “already experienced”; introduction of less experienced surgeons over duration of study | Primary: 3- and 5-year disease-free and overall survival  Operative  Short-term postoperative  Oncologic surrogate | Immediate postoperative stay |

*All studies were RCTs and compared laparoscopic to open surgery.

**Compared hand-assisted laparoscopic to open surgery.

Abbreviations: ASA: American Society of Anesthesiologists score; APR: abdominoperineal resection; AR: anterior resection; BMI: body mass index; FAP: familial adenomatous polyposis; IBD: inflammatory bowel disease; ITT: intention to treat analysis; lap: laparoscopic; LAR: low anterior resection; mets: tumor metastases; TME: total mesorectal excision.
